# A peptide‐display protein scaffold to facilitate single molecule force studies of aggregation‐prone peptides

**DOI:** 10.1002/pro.3386

**Published:** 2018-03-10

**Authors:** Ciaran P. A. Doherty, Lydia M. Young, Theodoros K. Karamanos, Hugh I. Smith, Matthew P. Jackson, Sheena E. Radford, David J. Brockwell

**Affiliations:** ^1^ Astbury Centre for Structural Molecular Biology, University of Leeds Leeds LS2 9JT United Kingdom; ^2^ School of Molecular and Cellular Biology, Faculty of Biological Sciences, University of Leeds Leeds LS2 9JT United Kingdom

**Keywords:** single molecule force spectroscopy, alpha‐synuclein, gamma‐synuclein, aggregation, amyloid, mass spectrometry

## Abstract

Protein aggregation is linked with the onset of several neurodegenerative disorders, including Parkinson's disease (PD), which is associated with the aggregation of α‐synuclein (αSyn). The structural mechanistic details of protein aggregation, including the nature of the earliest protein–protein interactions, remain elusive. In this study, we have used single molecule force spectroscopy (SMFS) to probe the first dimerization events of the central aggregation‐prone region of αSyn (residues 71–82) that may initiate aggregation. This region has been shown to be necessary for the aggregation of full length αSyn and is capable of forming amyloid fibrils in isolation. We demonstrate that the interaction of αSyn_71‐82_ peptides can be studied using SMFS when inserted into a loop of protein L, a mechanically strong and soluble scaffold protein that acts as a display system for SMFS studies. The corresponding fragment of the homolog protein γ‐synuclein (γSyn), which has a lower aggregation propensity, has also been studied here. The results from SMFS, together with native mass spectrometry and aggregation assays, demonstrate that the dimerization propensity of γSyn_71‐82_ is lower than that of αSyn_71‐82_, but that a mixed αSyn_71‐82_: γSyn_71‐82_ dimer forms with a similar propensity to the αSyn_71‐82_ homodimer, slowing amyloid formation. This work demonstrates the utility of a novel display method for SMFS studies of aggregation‐prone peptides, which would otherwise be difficult to study.

## Introduction

Studying protein aggregation is a significant challenge, owing to the fact that the aggregating species along the assembly pathway are often heterogeneous, transiently populated and evolve on an exponential timescale towards higher order, end point species.^1^, ^2^, ^3^ Early events in the aggregation cascade are consequently difficult to study but are of critical importance in enhancing our fundamental understanding of the aggregation process. Gaining a greater mechanistic and structural understanding of early events in aggregation is especially important for amyloid diseases where the identity of the toxic species is still unclear.^4^ Inhibition of early events in the aggregation cascade are thus attractive targets for preventative and curative treatments for these diseases.

Single molecule approaches using fluorescence,^5^, ^6^ or force spectroscopy using either the AFM^7^, ^8^, ^9^, ^10^, ^11^, ^12^, ^13^, ^14^, ^15^, ^16^, ^17^ or optical tweezers^18^ are potentially powerful methods to interrogate aggregating systems as they can be performed at low concentrations, limiting aggregation, and decreasing the rate of assembly. In SMFS, monomers of the aggregation‐prone species are immobilized to force sensors, separating the first protein–protein interactions from heterogeneous higher order species that complicate data analysis in other approaches. SMFS can yield information on both the strength of the interaction (and therefore off rates)^19^ and on the location of the interaction interface via measurement of the end‐to‐end length of the complex at rupture.^20^ Application of SMFS to the aggregation field, however, is challenging due to difficulties in deconvoluting nonspecific interactions from *bone fide* protein–protein interactions, both of which usually occur close to the surface.^21^ Separation of signal from noise is especially challenging when studying small aggregation‐prone proteins, such as Aβ42,^22^ or peptide fragments from larger proteins that are thought to drive aggregation such as the nonamyloid component (NAC) region of αSyn.^23^, ^24^ This is because these peptides can interact with inert surfaces and take part in multipartite interactions between the tip and surface. As the peptide end‐to‐end length is inherently short (<5 nm), these nonspecific interactions occur close to the surface. For experiments that extend a single protein (mechanical unfolding), some of these problems have been addressed by site‐specific immobilization onto passivated surfaces^25^ or by insertion of the protein of interest into a mechanically strong protein within a protein concatemer.^26^ For protein–protein interaction studies, Lyubchenko and coworkers have developed a “flexible nanoarray” method^27^, ^28^ whereby pairs of aggregation‐prone peptides are tethered onto an extensible linker at a defined separation, allowing dimerization events to be analyzed.^27^ Extending the tether using the AFM allows dissociation to be measured away from the surface. While allowing measurement of dissociation, this approach requires the synthesis of bespoke polyethylene glycol‐phosphoramidite linkers.^21^ Another approach developed by Vera and Carrión‐Vázquez^29^ used a disulfide‐linked polyprotein construct that allowed direct identification of single molecule dissociation events of the highly avid cohesin–dockerin complex. This method allows for internally controlled SMFS experiments on protein–protein interactions. It is, however, complex to implement, requires formation of a trimer and has not been used to analyze intermolecular interactions in a noncognate, aggregation‐prone system.

In this study, we report the development and validation of a novel, easy to implement method by which an aggregation‐prone region of a protein of interest is engineered into a loop of the mechanically stable monomeric carrier protein, protein L. The central NAC region (residues 71–82) of αSyn (part of the wider NAC region encompassing residues 61–95) was chosen to assess this display system as this hydrophobic, 12‐residue sequence (^71^VTGVTAVAQKT^82^V) is known to be both sufficient and necessary for aggregation^24^ and to form the core of fibrils of wild‐type αSyn.^30^ We show that protein L is a suitable scaffold protein, maintaining its structure and stability after insertion of guest sequences. SMFS experiments using an AFM tip and surface derivatized with the protein L scaffold containing αSyn_71–82_ yield rupture events with correlated dissociation force and unfolding distances. By contrast, protein L constructs containing a control (GS)_6_ linker or γSyn_71–82_ (the central NAC region derived from the less aggregation‐prone γSyn homolog of αSyn^31^, ^32^) yielded insignificant rupture events. Interestingly, an interaction is observed between αSyn_71–82_ and γSyn_71–82_ using SMFS in the pL scaffold, consistent with the postulated interaction between the full‐length proteins.^32^ We then use native electrospray ionization‐ion mobility spectrometry‐mass spectrometry (ESI‐IMS‐MS), which enables the detailed interrogation of individual species within a heterogeneous, assembling mixture,^33^, ^34^, ^35^, ^36^, ^37^ to validate these results. SMFS can thus yield novel insight into the early stages of amyloid formation by insertion of aggregation‐prone sequences of interest into the mechanically and thermodynamically stable scaffold, protein L.

## Results

### Design and characterization of a protein L scaffold

**Figure 1:**
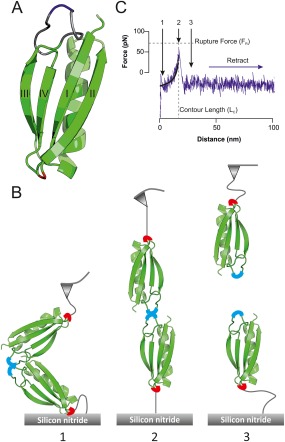
Using pL as a peptide‐presenting scaffold. (A) Structural model of the pL scaffold. The β3–β4 loop, peptide insert and Cys residue for immobilization are shown in gray, blue, and red, respectively. The β3–β4 loop was extended using SWISS‐MODEL and the PDB file 1HZ6.^38^ (B) Measuring chimeric pL dimer dissociation using the AFM. The derivatized AFM tip is brought into contact with a similarly derivatized surface (1) allowing dimerization via the guest peptide. The complex is then extended by retracting the cantilever, the force generated bends the cantilever and reaches a maximum (2) at complex dissociation [rupture force (*F*_R_)] prior to relaxation (3). (C) Typical force‐extension profile of the force induced dissociation of a mechanically resistant dimer. The positions of (1) dimerization, (2) dissociation, and (3) relaxation are shown. The contour length (*L*_C_) and *F*_R_ are measured for each dissociation event, the former parameter obtained by fitting the Worm Like Chain (WLC) model (black curve) to the profile (Materials and Methods section).

A scaffold protein should (a) allow the presentation of the amyloidogenic peptide in a defined geometry away from the surface; (b) be thermodynamically and mechanically stable after insertion of the sequence of interest, and (c) display minimal self‐aggregation. The IgG binding domain of protein L [Fig. 1(A)] fulfills these criteria. This protein comprises a four stranded β‐sheet packed against an α‐helix and has been shown previously to withstand the insertion of both folded and unfolded amino‐acid sequences into the β3‐β4 loop^39^ and to show mechanical resistance when extended via its termini.^40^ The scaffold shown in Figure 1(A) is based on protein L W47Y I60F (herein termed pL), a variant rationally designed to display enhanced mechanical strength.^41^ To ensure that the guest peptide sequence is sterically free from the pL host, the amyloidogenic peptide was inserted into the center of the β3‐β4 loop that had been extended by 13 residues (generating 55G…GGARGS…guest peptide…GSARGGG…^56^Y, Fig. S1, Supporting Information). Finally, a cysteine residue was introduced into the β1‐β2 loop (N14C) distal to the graft site. This allows the specific immobilization of the scaffold onto a substrate in a geometry that presents the guest sequence to the solution. When immobilized onto the AFM tip and surface, [Fig. 1(B)] this allows dimerization which, upon extension, would shear the pL N‐ and C‐terminal strands apart. This extension geometry has previously been shown to be mechanically robust.^40^, ^41^pL scaffolds presenting αSyn_71–82_ (Fig. S2, Supporting Information) and a 12‐residue nonaggregating control sequence [(GS)_6_, herein named GS] were constructed and each protein expressed and purified (Fig. S3, Supporting Information, Materials and Methods section). After verification of protein identity by ESI‐MS (Table SI, Supporting Information), the structural integrity of each construct was assessed by far‐UV CD and fluorescence emission spectroscopy. These experiments verified that all three proteins exhibited CD‐spectra consistent with a mixed α/β topology similar to that reported for wild‐type protein L^42^ [Fig. S4(A), Supporting Information]. Similarly, fluorescence emission spectra which exhibit a red shift in their maxima upon denaturant‐induced unfolding were observed for all proteins [Fig. S4(B), Supporting Information]. As expected for natively folded variants of protein L, thermal denaturation monitored by far‐UV CD revealed co‐operative unfolding transitions at a similar temperature for pL GS and pL αSyn_71–82_ [*T*_m_ values of 45.5 and 46.6°C, respectively, Fig. S4(C, D), Supporting Information].

**Figure 2:**
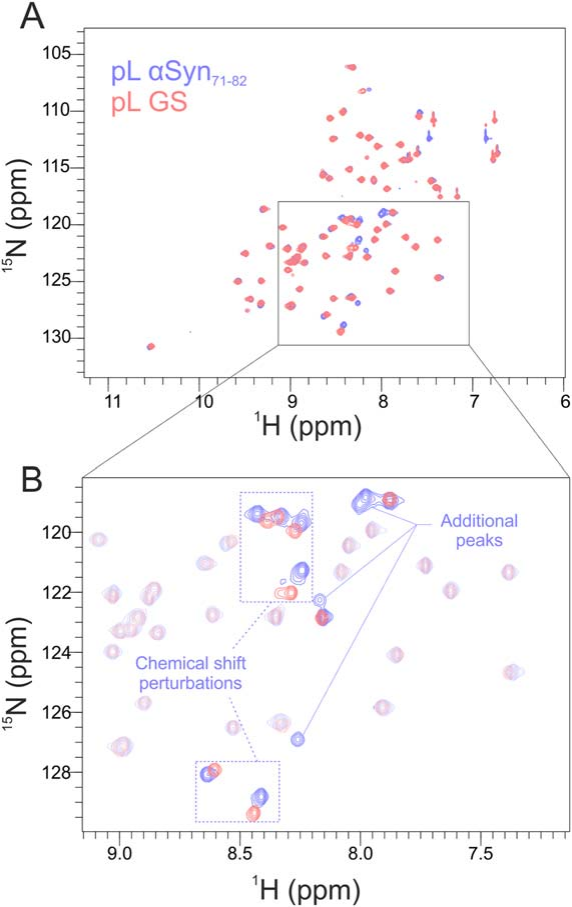
pL αSyn_71–82_ and pL GS have similar structures as revealed by HSQC NMR. The data presented shows 600 MHz ^1^H‐^15^N HSQC‐NMR spectra of 15N labeled pL αSyn_71–82_ and pL GS. (A) Overlaid spectra of pL GS (red peaks) and pL αSyn_71–82_ (blue peaks). Both spectra have dispersed and well‐defined peaks characteristic of folded proteins. Additional peaks observed for pL αSyn_71–82_ at ∼8 ppm in the ^1^H dimension most likely arise from residues in the inserted peptide in an unfolded dynamic conformation. (B) Expanded region highlighting additional peaks that are observed for pL αSyn_71–82_. A small number of residues showing chemical shift perturbations are also highlighted (blue dashed boxes). Shifts arise because residues form part of, or are close to, the β3‐β4 loop.

As a more sensitive probe for structural perturbations, the ^1^H‐^15^N heteronuclear single quantum coherence spectroscopy (HSQC) NMR spectra of pL αSyn_71–82_ and pL GS were acquired (Fig. 2). Both spectra show similarly dispersed, well defined peaks of similar intensity, characteristic of stably folded proteins [Fig. 2(A)]. Five additional main chain peaks were observed for pL αSyn_71–82_ compared with pL GS [Fig. 2(B)], likely due to the chemical shift redundancy of the glycine residues in the (GS)_6_ linker and extended loop. Most of the additional peaks for pL αSyn_71–82_ are observed at ^1^H chemical shifts that are diagnostic of an unfolded protein conformation (∼8.0 ppm), suggesting that these peaks indeed arise from unstructured αSyn_71–82_. Overall, the CD, fluorescence, and NMR data show that grafting a 12‐residue peptide sequence into the extended β3‐β4 loop does not perturb the structure or stability of pL, validating its use a display scaffold for SMFS experiments.

### Dimerization of the αSyn central NAC region detected by SMFS

**Figure 3:**
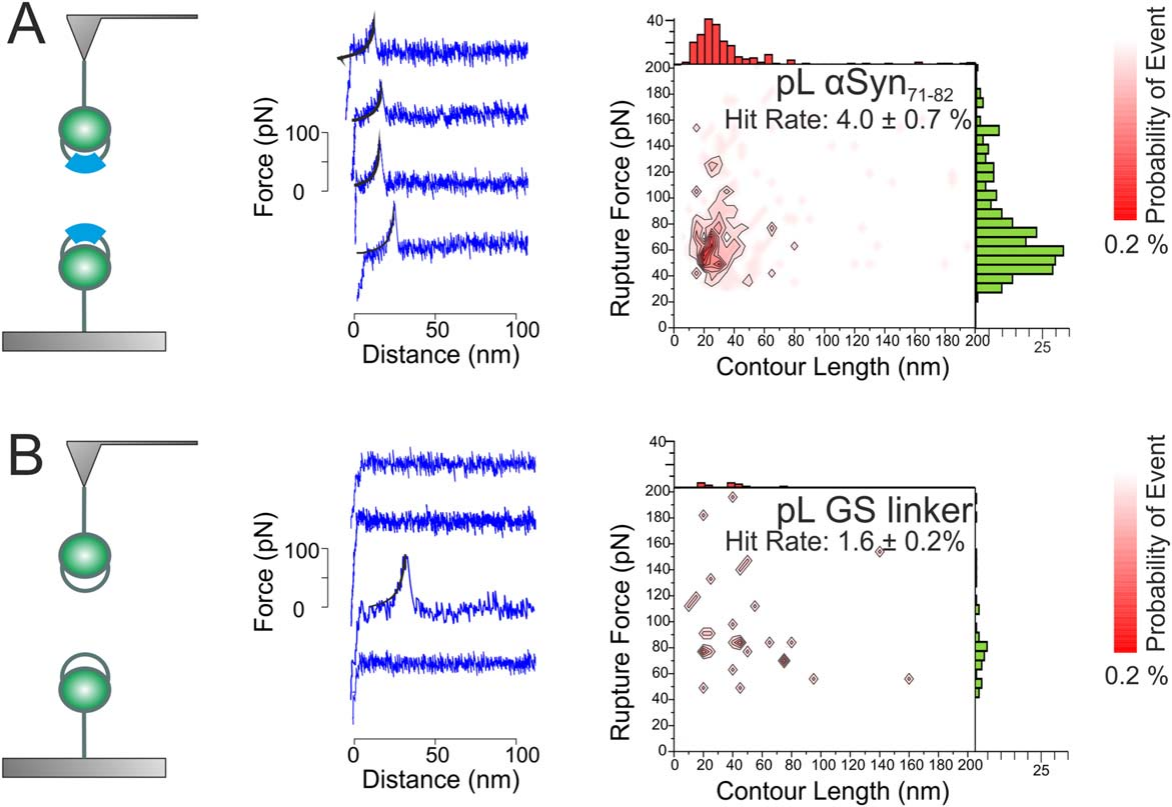
SMFS can detect dissociation events for pL αSyn_71–82_ but not pL GS. Schematic (left), sample force extension profiles (middle), and a *F*_R_ versus *L*_c_ scattergram (right, rendered as a contour plot and showing the associated *L*_c_ and *F*_R_ frequency‐histograms above and to the side, respectively for (A) pL αSyn_71–82_ and (B) pL GS dissociation events). A WLC (black line) is fitted to each force‐extension profile. The contour plot for pL αSyn_71–82_ shows a “hotspot” of reproducible and correlated *L*_C_ and *F*_R_ values. No “hotspot” is visible for pL GS dissociation events. The total number of force‐retract cycles (total triplicate) are 8000 and 2500 for pL αSyn_71–82_ and pL GS dimerization interactions, respectively.

To determine whether the association of αSyn_71–82_ could be detected in the context of the protein L scaffold using AFM‐based SMFS methods, the AFM probe and surface were each decorated with either pL αSyn_71–82_ or pL GS (Materials and Methods). After mounting in the AFM, force‐extension profiles were accumulated. After filtering (Materials and Methods section), traces which showed a characteristic single molecule “saw tooth” profile [Fig. 1(C)] were binned for analysis. The number of these events relative to the total number of approach‐retract cycles (in triplicate) was then used to calculate a ‘hit rate’ which reports on the probability of measuring the rupture (and therefore the presence) of a protein–protein interaction. As shown in Figure 1(C), for each force‐extension profile, the force at rupture (*F*_R_) of the interaction and the end‐to‐end length of the dimer (*L*_c_) at rupture was measured. To obtain *L*_c_, force‐extension profiles were fitted to the WLC model [Materials and Methods section, Eq. (1)]. Finally, to assess whether the observed dissociation events produced reproducible and correlated values of *L*_C_ and *F*_R_, contour plots were generated (Fig. 3).

Comparison of the force‐extension profiles and contour plots obtained for pL αSyn_71–82_ and pL GS [Fig. 3(A, B)] revealed that the dissociation of pL αSyn_71–82_, but not pL GS, can be detected and quantified using SMFS. Force‐extension profiles for pL αSyn_71–82_ typically produced a single sawtooth [Fig. 3(A)], as expected for an extensible chain^43^ with correlated *F*_R_ and *L*_C_ values (57 ± 1 pN and 22 ±1 nm, respectively) and a hit‐rate of 4.0 ± 0.7%. By contrast, force‐extension profiles for pL GS were generally featureless [Fig. 3(B)]. For this protein, the frequency of binned events was lower (1.6 ± 0.2%) with no ‘hotspot’ (diagnostic of a specific interaction) in the contour plot. These data indicate that SMFS can detect intermolecular interactions between amyloidogenic peptides. The modal rupture force is surprisingly large given the expected transient nature and the relatively small interaction surface of the 12‐residue αSyn peptide (*F*_R_ = 57 pN, at a retraction velocity of 1000 nms^−1^). The *F*_R_ measured here is, however, similar to that measured for full length αSyn^14^, ^16^, ^17^ and other protein–protein interactions mediated by short peptide sequences.^44^

### Dynamic force spectroscopy of the αSyn Central NAC region

**Figure 4:**
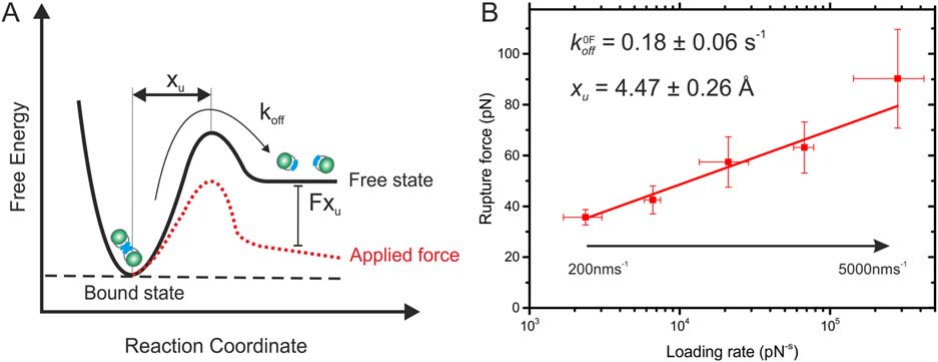
Using dynamic force spectroscopy to investigate αSyn_71–82_ dissociation. (A) Schematic representation of an unbinding energy landscape. The dotted line depicts the tilted energy landscape under force. *x*_U_ is depicted as the distance from the bound energy well to the transition state. $k_{off}^{0F}$ is shown as the stochastic process of crossing the transition energy barrier. (B) Dynamic force spectrum of αSyn_71–82_ dissociation. Data (mean of the triplicate datasets, error bars are the standard deviation) are fitted to the Bell‐Evans model^45^, ^46^ to obtain values $k_{off}^{0F}$ and *x*_U_ (shown inset). The errors for $k_{off}^{0F}$ and *x*_U_ were calculated by manual bootstrapping. The total number of approach retract cycles (total triplicate) for pL αSyn_71–82_ dimer dissociation events are 5200, 5500, 6500, 6000, and 5500 for pulling speeds 200, 500, 1000, 3000, and 5000 nms^−1^, respectively.

Despite the small size of the peptide fragment αSyn_71–82_ under study here, the data demonstrate that interaction between these sequences is sufficiently strong to be detected by SMFS. In order to characterize this dimer interaction further, dynamic force spectroscopy (DFS) was carried out allowing parameters of the unbinding energy landscape to be calculated [Fig. 4(A)]. The SMFS experiments were carried out at pulling velocities ranging from 200 to 5000 nms^−1^ [Fig. 4(B)]. After calculation of the loading rate at rupture [Materials and Methods section Eq. (2)], the dissociation rate constant in the absence of force ($k_{off}^{0F}$) and the ‘distance’ between the dissociation energy barrier and the bound ground state (*x*_u_) was calculated using the Bell‐Evans model^45^ ^,46^ [Materials and Methods section, Eq. (3)]. The dissociation rate constant for pL αSyn_71–82_ dimers ( $k_{off}^{0F}$ = 0.18 s^−1^, lifetime ∼6 s) is comparable to the dissociation rate constant of full length αSyn (0.25 s^−1^ at pH 2.7 and 0.74 s^−1^ at pH 3.7 measured by SMFS).^9^ The finding that the lifetimes of dimers of full‐length αSyn and αSyn_71–82_ dimers are of comparable magnitude suggests that residues 71–82 play a key role in the stability of dimeric species formed from the intact protein.

### SMFS identifies a novel interaction between αSyn_71–82_ and its homolog γSyn_71–82_

While the presence or absence of SMFS rupture peaks for dimers of pL αSyn_71–82_ and pL GS, respectively suggests that the former protein self‐interacts, it does not report on the specificity of this interaction. To address this question, we inserted the central NAC domain of another member of the synuclein family, γSyn, into the pL scaffold. The central NAC sequences of α‐ and γ‐Syn differ at five positions (αSyn: ^71^VTGVTAVAQKTV_82_ and γSyn: _71_VSSVNTVATKTV^82^) and these differences (Fig. S2, Supporting Information) may play a role in the greater amyloidogenic propensity observed for αSyn.^31^, ^32^

**Figure 5:**
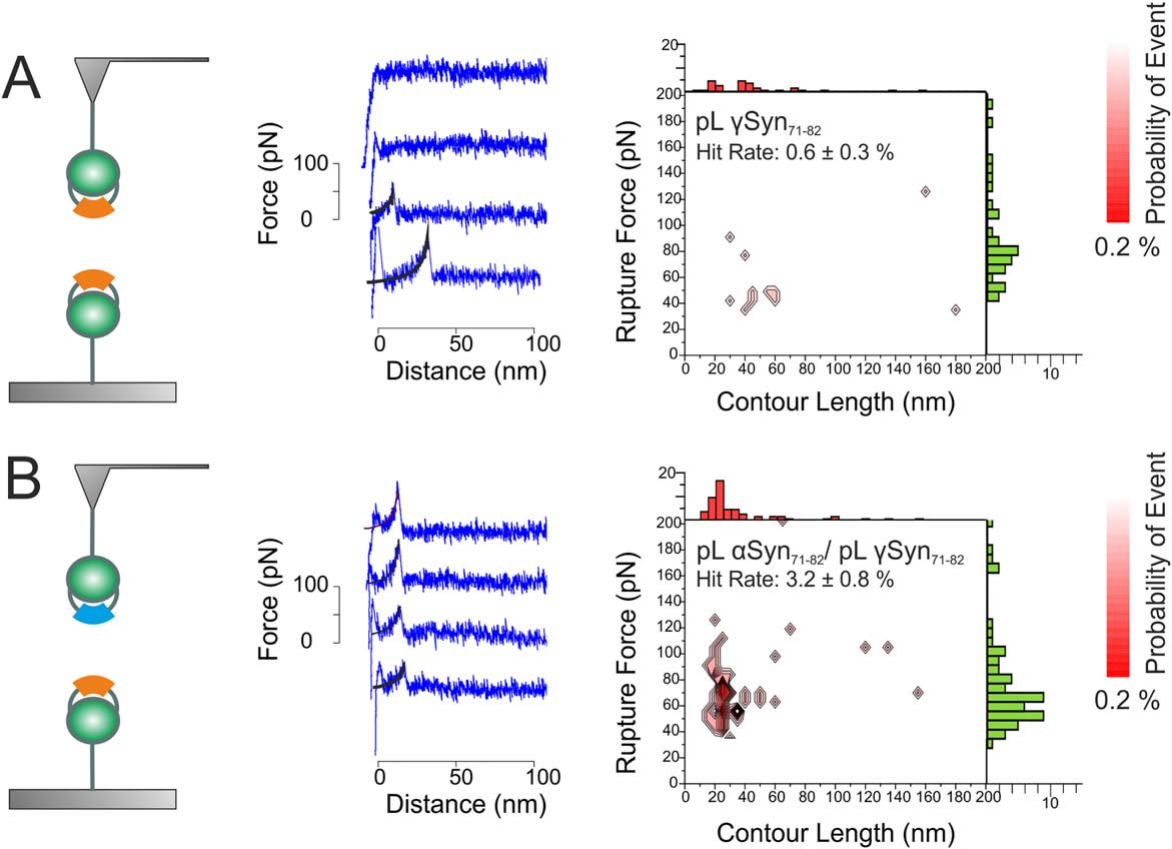
SMFS can detect dissociation events for pL αSyn_71–82_: pL γSyn_71–82_ heterodimers but not pL γSyn_71–82_ homodimers. Schematic (left), sample force extension profiles (middle) and a *F*_R_ versus *L*_c_ contour plot (right), showing the associated *L*_c_ and *F*_R_ frequency‐histograms above and to the side, respectively for (A) pL γSyn_71–82_ homodimer dissociation events and (B) pL αSyn_71–82_:pL γSyn_71–82_ heterodimer dissociation events. WLC fits (black line) are fit for force extension profiles. While no “hotspot” is visible for the dissociation of pL γSyn_71–82_ homodimers, the contour plot for dissociation of the heterodimer shows a “hotspot” of reproducible and correlated *L*_C_ and *F*_R_ values. The modal *L*_C_ and *F*_R_ were calculated to be 19 ± 1 nm and 53 ± 1 pN, respectively. The total number of approach retract cycles (total triplicate) for pL γSyn_71–82_ homodimerization and αSyn_71–82_/γSyn_71–82_ heterodimerization interactions are both 2500.

After construction, purification and characterization of pL γSyn_71–82_ (Figs. S3, S4, Table SI, Supporting Information), SMFS was used to probe homodimeric (pL γSyn_71–82_ on the AFM tip and surface) and heterodimeric (pL γSyn_71–82_ on the AFM tip and pL αSyn_71–82_ on surface) dissociation events. Surprisingly, the force‐extension profiles and contour plots for putative homodimeric pL γSyn_71–82_ dissociation [Fig. 5(A)] show no evidence for formation of a force‐resistant structure (hit rate = 0.6 ± 0.3%, no ‘hot spot’ evident). By contrast, the dissociation of pL γSyn_71–82_:pL αSyn_71–82_ heterodimers was readily detected using SMFS yielding comparable *L*_C_ and *F*_R_ values (19 ± 1 nm and 53 ± 1 pN, respectively) to that of the αSyn_71–82_ homodimeric interaction, albeit at a lower frequency of detection (3.2 ± 0.8%). Importantly, the possibility that these observations arise from differences in immobilization efficiencies between each scaffold was ruled out by sequential experiments that used the same derivatized tip but different substrates (Fig. S5, Supporting Information). The results thus portray the first observation of a heterodimeric interaction in an aggregating system by SMFS and also the first observation that the central NAC region of αSyn is sufficient to interact with the corresponding sequence of its homolog γSyn. Uversky *et al*. have shown previously that full length αSyn fibrillation is inhibited by the presence of γSyn.^32^ One possible interpretation is that this inhibitory effect is mediated by the interaction between the central NAC regions of these proteins.

### Native mass spectrometry of pL constructs

**Figure 6:**
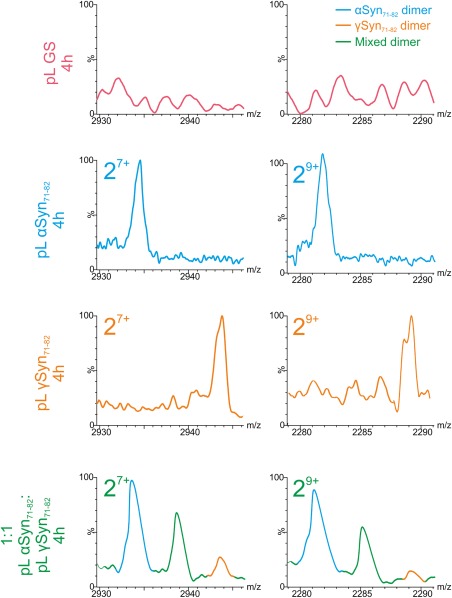
Dimeric species of chimeric pL constructs analyzed by ESI‐MS. ESI‐mass spectra showing two different charge states (7+ and 9+) of the dimeric species formed after 4 h incubation of pL GS (pink), pL αSyn_71–82_ (blue), pL γSyn_71–82_ (orange), and 1:1 pL αSyn_71–82_ and pL γSyn_71–82_ (heterodimer colored green). The numbers denote the oligomer order, with the positive‐charge state of ions in superscript. The spectra of pL GS shows the absence of dimer after 4 h. The difference in mass between pL αSyn_71–82_ and pL γSyn_71–82_ allows pL αSyn_71–82_ and pL γSyn_71–82_ homodimers and heterodimeric species to be readily discerned from one another.

SMFS shows that the central NAC regions of α‐ and γSyn are able to dimerize and be of sufficient kinetic stability to measure their dissociation using force spectroscopy. To verify this observation, ESI–MS was used as an orthogonal method to detect dimerization. This soft ionization method enables preservation of noncovalent interactions in the gas phase and allows resolution of heterogeneous species that differ in mass or charge state and, when coupled to ion mobility spectrometry (ESI–IMS–MS), cross‐sectional area.^47^ These experiments were performed using 100 μ*M* pL constructs in 100 m*M* ammonium acetate pH 6.8, and to measure formation of heterodimers, equal volumes of each sample were mixed. ESI–MS data were then accumulated immediately (*t* = 0) or after quiescent incubation for 4 h at 25°C. Comparison of the ESI mass spectra for pL GS, pL αSyn_71–82_, pL γSyn_71–82_, and 1:1 pL αSyn_71–82_:pL γSyn_71–82_ showed that all pL constructs were initially monomeric upon dilution (*t* = 0, Fig. S6, Supporting Information). In accord with the SMFS experiments described above, pL GS remained monomeric after incubation. In contrast, dimers, in addition to monomers, were observed for pL γSyn_71–82_ alone, with monomers–trimers present for pL αSyn_71–82_ and for mixtures of 1:1 pL αSyn_71–82_:pL γSyn_71–82_ (Fig. S6, Supporting Information). For each charge state of the heterodimeric species, the difference in the molecular masses of pL αSyn_71–82_ and pL γSyn_71–82_ (10,391 and 10,423 Da, respectively) is sufficient to resolve three peaks representing homodimers of pL αSyn_71–82_ and pL γSyn_71–82_ (at m/z values for the 9+ charge state centered on ∼2282 and 2289, Fig. 6) and a heterodimer (m/z centered on ∼2285) corroborating the observation of a heterodimer in SMFS experiments. Interestingly, despite mixing an equal concentration of each variant, the relative intensity of each dimer species differs and mirrors the hit rate observed by SMFS experiments (from highest to lowest apparent dimer population: αα > αγ > γγ). In the mixed samples, peaks from monomeric proteins (Fig. S6, Supporting Information) were of similar intensities, suggesting that the two peptides ionize with similar efficiency. This observation suggests that the intensities of dimer peaks indeed reflect the relative affinity of the different dimeric species.

To preclude the possibility that the interactions observed by SMFS and ESI‐MS arise from the pL scaffold, the ESI–MS experiments were repeated using αSyn_71–82_ and γSyn_71–82_ as synthetic peptides. ESI–IMS–MS driftscope plots of the peptides alone and in a 1:1 mixture showed the presence of monomeric to tetradecameric species (Fig. S7, Supporting Information). The higher order of species observed for the peptide‐only variants suggests that packing restraints of the scaffold disfavors larger oligomer formation. Despite these differences, homodimers and heterodimers were formed upon mixing equimolar αSyn_71–82_:γSyn_71–82_, confirming that αSyn_71–82_ and γSyn_71–82_ sequences interact independently of the pL scaffold.

### Characterizing the aggregation of pL constructs

**Figure 7:**
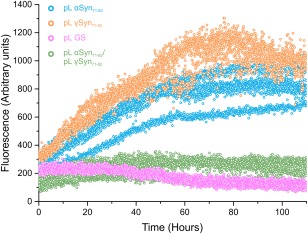
ThT fibril formation assays show that pL αSyn_71–82_ inhibits the aggregation of pL. γSyn_71–82_. ThT fluorescence assay showing the normalized fluorescence over time of pL αSyn_71–82_ (blue), pL γSyn_71–82_ (orange), pL GS (pink), and the 1:1 mix of pL αSyn_71–82_ and pL γSyn_71–82_ (green). Proteins were incubated at 37°C, 600 rpm at a final concentration of 100 μ*M* (α/γ mix at 50 μ*M* of each protein).

We have shown that the central NAC regions of αSyn and γSyn are sufficient to allow the formation of transient homo‐ and hetero‐dimers and that this interaction is preserved when these short sequences are inserted into the pL scaffold. To investigate whether these pL‐peptide chimeras are able to form amyloid fibrils, we examined the amyloid growth kinetics of pL αSyn_71–82_ and pL γSyn_71–82_ using thioflavin T (ThT) fluorescence (Fig. 7). In addition, as full length γSyn has previously been shown to inhibit the kinetics of αSyn aggregation,^32^ we also measured the amyloid growth kinetics of a 1:1 mixture of pL αSyn_71–82_ and pL γSyn_71–82_ to test whether this inhibitory interaction was mediated by the central NAC region. The end point of ThT fluorescence emission intensity was similar for both pL αSyn_71–81_ and pL γSyn_71–82_ (785 ± 122 and 929 ± 83 arbitrary units, respectively after 100 h) while the mixed sample appeared unable to aggregate as studied by this assay (Fig. 7). pL GS did not aggregate into ThT‐positive species. Similar experiments performed on the synthetic peptides also showed that a 1:1 mixture of αSyn_71–81_ and γSyn_71–82_ resulted in a significant increase in lag‐time [2.5× and 5.7× increase when compared with αSyn_71–82_ and γSyn_71–82_ alone, respectively (Fig. S8, Supporting Information)]. These data suggest that the heterodimeric interaction identified by SMFS and ESI–MS inhibits aggregation. This supports the finding that the inhibitory effect reported for full length γSyn on the aggregation of αSyn is mediated by the interaction between their central NAC regions. It is important to note however, that the SMFS and ESI–MS experiments were carried out in different buffer conditions due to the buffer limitations in ESI–MS experiments. It is widely recognized that ionic strength and pH affect the rates and the dominating mechanisms in amyloid formation.^48^, ^49^, ^50^ The inhibitory effect that γSyn_71–82_ exerts on αSyn_71–82_ was, however, also observed in aggregation assays performed in ESI–MS buffer conditions (Fig. S9, Supporting Information).

## Discussion

In this study we describe the generation, characterization and proof‐of‐concept of a novel approach to facilitate the investigation of interactions between amyloidogenic peptides using SMFS. Using protein L as a display system, we were able to interrogate the specific interactions between synuclein sequences and to minimize the contributions of nonspecific events that often occur at the surface‐proximal regions of force‐distance curves.^51^ Our approach also restricts the application of force to a defined geometry, giving more confidence that specific dimer interactions are being interrogated in a consistent manner.

Importantly, we have shown that protein L maintains sufficient stability upon insertion of amyloidogenic sequences to fold to a native state. This is significant as the ability of chimeric pL constructs to fold to a native state is integral to the ability to display peptide sequences to reduce nonspecific tip‐surface interactions.

Whilst the aim of this study was to assess the potential of a scaffold display for SMFS experiments, the study has also yielded intriguing observations on the dimerization between αSyn and its less aggregation‐prone homolog γSyn, by both SMFS and native ESI‐mass spectrometry. First, DFS of the dissociation of pL αSyn_71–82_ dimers revealed a lifetime that is similar to that of full length αSyn (6 and 4 s for pL αSyn_71–82_ and full length αSyn, respectively) measured using the same technique.^9^ It is important to note, however, that previous SMFS experiments on full‐length αSyn were conducted at acidic pH where the full length protein is closer to its pI and therefore more prone to aggregation.^7^, ^9^, ^12^, ^14^, ^16^, ^17^ The experiments carried out here were conducted at a more physiological pH (pH 7.5) and are therefore more applicable to the conditions in which the protein self‐associates *in vivo*.

To investigate the specificity of the dimer interaction, the dissociation of pL γSyn_71–82_ homodimers and pL αSyn_71–82_:pL γSyn_71–82_ heterodimers was also investigated by SMFS. Surprisingly, no reproducible correlation between F_R_–L_c_ values was found for pL γSyn_71–82_ homodimers, suggesting that under the conditions used, dimerization did not occur or that these dimers dissociated more quickly or at a force that cannot be detected in this experiment. By contrast, dissociation of the αSyn_71–82_: γSyn_71–82_ heterodimer yielded AFM data that were similar to those obtained for pL αSyn_71–82_ homodimers. Despite this similarity, a population of pL αSyn_71–82_:pL γSyn_71–82_ dimers inhibits aggregation in the context of both chimeric pL constructs and synthetic peptides equivalent to the central NAC region in isolation. This suggests that the mechanism by which full length γSyn inhibits the aggregation of αSyn^32^ may be mediated by the central NAC region of the proteins. As both αSyn and γSyn are highly expressed in many of the same cells of the brain,^52^ it may be postulated that heterogeneous αSyn and γSyn dimers occur *in vivo*, which may chaperone αSyn away from aggregation under normal cellular conditions. The importance of this region to the aggregation of αSyn has been highlighted in a recent study,^53^ which shows that αSyn_71–82_ derived peptides bind full‐length αSyn, and that modified peptides can increase its aggregation.

Together these observations provide further support for the central role of the NAC sequence in the aggregation of full length αSyn. The ability of βSyn to inhibit the aggregation of αSyn, challenges this notion, as this homolog has no NAC region but forms transient interactions with αSyn up‐ and down‐stream from the NAC sequence.^32^, ^54^ It is becoming clear that this intrinsically disordered protein displays conformational plasticity and that long range intra‐ and inter‐chain interactions between the termini of this protein, which can be disrupted by changes in pH or mutation, may modulate the interaction potential of NAC.^55^, ^56^, ^57^ The development of a facile method that will allow the relative frequency and dissociation rate of these, and other interactions, such as that reported here, may help to understand the early events in the etiology of αSyn aggregation relevant to Parkinson's disease.

## Materials and Methods

### Expression and purification of pL constructs

pL constructs (Fig. S1, Supporting Information) encoded on pET23a plasmids were expressed in *Escherichia coli* BL21 (DE3) cells. Briefly, overnight starter cultures were used to inoculate 10 × 1 L LB medium supplemented with 100 μg/mL ampicillin and grown to an optical density at 600 nm = 0.6. Protein expression was then induced by addition of IPTG to a final concentration of 1 m*M*. The cultures were allowed to grow for a further 4 h before harvesting. Cell pellets were resuspended in lysis buffer [20 m*M* Tris.HCl pH 8.0, 300 m*M* NaCl, 20 m*M* imidazole, 2 m*M* DTT, 0.025% (w/v) sodium azide, 1 m*M* PMSF, 2 m*M* benzamidine, 0.15% (v/v) Triton X100, 20 μg/mL DNase, and 100 μg/mL lysozyme], homogenized and further lysed by cell disruption at 30 K PSI. Cell debris was removed by centrifugation at 18,000*g* for 30 min. The proteins were purified by affinity chromatography using a 5 mL His‐Trap FF column (GE Healthcare) equilibrated with lysis buffer (without Triton X100, DNase, and lysozyme) and eluted by a stepped gradient (25, 50, and 100%) of 20 m*M* Tris.HCl pH 8.0, 300 m*M* NaCl, 250 m*M* imidazole, 2 m*M* DTT, 0.025% (w/v) sodium azide, 1 m*M* PMSF, 2 m*M* benzamidine. pL complexes were further purified using size exclusion chromatography (HiLoadTM 26/60 Superdex 75 prep grade column, GE Healthcare) equilibrated with 25 m*M* Tris.HCl, 300 m*M* NaCl, 2 m*M* DTT, pH 8. The protein was then dialyzed into 20 m*M* HEPES, pH 7.5 (for pL SMFS experiments) and flash frozen with liquid N_2_. The presence, purity and the correct mass of proteins were confirmed by ESI–MS.

### Thioflavin T (ThT) aggregation assays

One hundred micromolar pL constructs (in 20 m*M* HEPES, 20 μ*M* ThT, pH 7.5) were incubated at 37°C shaking at 600 rpm in a BMG Labtech FLUOstar optima plate reader in Corning 96‐well flat bottom assay plates. Plates were sealed with StarSeal advanced polyolefin film (Starlab, Hamburg, Germany). To monitor growth kinetics, samples were excited at 444 nm and the fluorescence emission was monitored at 480 nm with a gain of 1450. Data were accumulated every 6.67 min over a period of >100 h.

### Synuclein peptides and Thioflavin T (ThT) fluorescence

αSyn_71–82_ and γSyn_71–82_ peptides were purchased from Genscript, NJ at >99% purity. All peptides were N‐terminally acetylated and C‐terminally amidated. Peptides were dissolved in 100% (v/v) HFIP (hexoflouroisopropanol) at 450 μ*M* and dispensed into Corning 96‐well flat bottom assay plates. Fifty microliter was dispensed into wells, and the HFIP was left to evaporate. The dry peptide in the well was dissolved into 100 μL 20 m*M* HEPES, 20 μ*M* ThT, pH 7.5 to give a concentration of 225 μ*M* peptide. Incubations were carried out at 37°C shaking at 600 rpm. Plates were sealed with StarSeal advanced polyolefin film from Starlab, Hamburg, Germany. The fluorescence of the samples were excited at 444 nm and the fluorescence emission was monitored at 480 nm on a BMG Labtech FLUOstar optima plate reader with a gain set at 1450. Where normalized data is presented, it has been processed on the plate reader software and normalized (after buffer subtraction) between 0 and 100. Lag time analysis was carried out by manually fitting a linear regression of the steepest exponential region of the ThT curves on Origin Pro 9.1 software. The fit was extrapolated to calculate the x‐intercept (quoted lag times).

### ^1^H‐^15^N HSQC NMR spectroscopy

^1^H‐^15^N HSQC spectra of 400 μ*M* pL αSyn_71–82_ and pL γSyn_71–82_ (20 m*M* HEPES buffer, 10% (v/v) D_2_O and 0.02% (w/v) sodium azide, pH 7.5) were recorded on an AVANCE III Bruker spectrometer (600 MHz) equipped with a cryogenic probe. Spectra were processed in NMRPipe and analyzed in CCPN analysis.

### Negative stain transmission electron microscopy (TEM)

Protein samples were pipetted onto the surface of carbon coated copper grids and stained with 1% (w/v) uranyl acetate. Images were taken on an FEI T12 electron microscope.

### AFM surface and cantilever derivatization

AFM surface and cantilever functionalization was performed as described previously.^44^ Prior to an experiment, AFM probes and surfaces were immersed in ∼1 mL chloroform containing 20 μL of 250 m*M* maleimide‐PEG‐NHS ester (MW 3400 Da, Nanocs, NY) in DMSO and incubated at room temperature for 1 h. The PEG linkers used in this study are polydisperse with masses ±5% of the stated mass. AFM probes and surfaces were then washed with chloroform and dried with a stream of N_2_ gas.

One hundred microliter pL constructs (50 µ*M* in 20 m*M* HEPES, pH 7.5) containing engineered cysteine residues were deposited over the functionalized surface and AFM probe and left to incubate in a covered container for 30 min at room temperature. All proteins analyzed by SMFS were immobilized to functionalized probes and surfaces in the presence of 1 m*M* TCEP (*tris*(2‐carboxyethyl)phosphine) to limit disulfide crosslinkage between proteins. Unreacted protein was then washed from the surface and AFM probe with excess reaction buffer.

### Force spectroscopy

All AFM measurements were conducted on an Asylum MFP‐3D microscope using Si_3_N_4_ cantilevers with nominal spring constants of 30 pN nm^−1^ (Bruker MLCT). For each cantilever used, the spring constant was determined using the thermal method^58^ using Asylum software. The approach speed was kept constant at 2 µm s^−1^. A retraction speed of 1 μm s^−1^ was used for SMFS experiments that probed the dimerization of scaffolds. Typically force maps of 500 force‐distance curves were taken over a 20 µm^2^ area. One dataset typically comprised four force maps (2000 force distance curves). All experiments were conducted in 20 m*M* HEPES pH 7.5 at room temperature.

All force spectroscopy data were analyzed using IGOR pro 6.32A with an Asylum Research extension (MFP3DXop v30). A force‐extension profile was binned for analysis when single characteristic, parabolic WLC events were observed. For each profile, the force (*F*_R_) and contour length (*L*_C_) at rupture was measured. To obtain *L*_C_, each force‐extension profile was manually fit to a WLC model^59^ [with a fixed persistence length of 0.4 nm, Eq. (1)].

(1) $$F\left(x\right)=\frac{k_{B}T}{p}{\left(0.25\left(1-\frac{x}{L_{C}}\right)\right)}^{2}-\;0.25+\;\frac{x}{L_{C}}$$

where F is the entropic restoring force, x is the extension, p is the persistence length, $k_{B}$ is the Boltzman constant, *T* is the absolute temperature, and *L*_C_ is the contour length. Hit rates were calculated as the number of hits as a percentage of total approach‐retract cycles. The average values over multiple experiments are quoted. The errors on hit rates are the standard deviation between experiments.

Single Gaussian distributions were fit to frequency histograms in order to determine the most probable *F*_R_ and *L*_C_ for each retraction velocity investigated. For each pulling velocity used, data were collected in triplicate (using a freshly prepared cantilever for each repeat).

To obtain a dynamic force spectrum (DFS), force‐extension data were collected using retraction speeds between 200 and 5000 nms^−1^. To analyze these data, loading rates were calculated by fitting a WLC model to the rising edge of each unbinding profile when plotted as force versus tip‐sample separation. The instantaneous gradient of this fit at rupture (WLC_slope_) was calculated by inserting the derived contour length and extension at rupture into a differentiated form of the same equation [Eq. (2)]. The loading rate at rupture was then obtained by multiplying this value by the retraction velocity.

(2) $${WLC}_{slope}=\frac{k_{B}T}{p}\left(\frac{1}{2L_{c}{\left(1-\frac{x}{L_{c}}\right)}^{3}}\right)+\frac{1}{L_{c}}$$

where *p* is the persistence length, *L*_C_ is the contour length, *x* is the extension, $k_{B}$ is the Boltzmann constant, and *T* is the temperature.

The natural logarithm of the mean loading rate (Ns^−1^) at each velocity was plot against the mean rupture force (*N*), which gives a linear relationship. The Bell‐Evans model^45^ [Eq. (3)] was rearranged to use the gradient of the linear fit to calculate the distance from the transition state (*x*_u_) and the *y*‐intercept for the off rate at zero force ( $k_{off}^{0F}$)

(3) $$F_{R}=\left(\frac{k_{B\;}T}{x_{u}}\right)\ln\left(\frac{r_{f}\;x_{u}}{k_{off}^{0F}k_{B}T}\right)$$

where *k*_B_ is the Bolzmann's constant, *T* is the temperature (in Kelvin), *r*_f_ is the rate at which force is loaded onto the complex or loading rate, *x*_u_ is the distance from the low energy state to the transition state and $k_{off}^{0F}$ is the spontaneous unfolding or unbinding rate in the absence of force.

### Mass spectrometry

A Synapt HDMS quadrupole‐time‐of‐flight mass spectrometer (Waters, Manchester, UK), equipped with a Triversa NanoMate (Advion Biosciences, Ithaca, NY) automated nano‐ESI interface, was used for these analyses. The instrument has a traveling ‐wave IMS device situated between the quadrupole and the time‐of‐flight analyzers.

αSyn and γSyn peptide samples and pL chimeric constructs (100 μ*M* final concentration in 100 m*M* ammonium acetate buffer, pH 6.8) were analyzed using positive mode nanoESI with a capillary voltage of 1.7 kV and a nitrogen nebulizing gas pressure of 0.8 psi. The following instrumental parameters were used: cone voltage 30 V; source temperature 60°C; backing pressure 3.2 mBar; ramped traveling wave height 7–20 V; traveling wave speed 300 ms^−1^; IMS cell pressure 0.55 mBar. Data were acquired over the range m/z 500–6000. Data were processed by use of MassLynx v4.1 and Driftscope v2.4 software supplied with the mass spectrometer. Mass calibration was achieved using cesium iodide solution, prepared by dissolving the compound in 50% (v/v) water/isopropanol to a concentration of 2 mg/mL.

### Circular dichroism (CD)

Far UV (190–260 nm) CD spectroscopy was performed on 50 μ*M* pL constructs (25 m*M* sodium phosphate buffer, 2 m*M* DTT, pH 8.0) in a 1 mm path length cuvette (Hellma) using a Chirascan™‐plus CD Spectrometer (Applied Photophysics, UK). CD spectra were acquired using a 1 nm bandwidth at room temperature, 1 s time step. Three scans were taken per sample.

For thermal denaturation experiments, a temperature gradient from 20 to 90°C in 1°C steps was performed. Protein samples were incubated for 180 s at each temperature before CD spectra were taken as above. The thermal melt data were analyzed on Photophysics Global3 software.

### Fluorescence spectroscopy

Intrinsic tryptophan emission spectra of 50 μ*M* pL constructs (25 m*M* sodium phosphate buffer, 2 m*M* DTT, pH 8.0) were measured on a Photon Technology International fluorometer (Ford, West Sussex, UK). Excitation and emission slit widths were set to 1 and 2 nm, respectively. Proteins were excited at 280 nm and emission spectra were recorded at 290–400 nm. Spectra were recorded of three replicates. All data were normalized to the unfolded state. The unfolded state was recorded in the same conditions as above in the presence of 8*M* urea.

## Conflict of Interest

The authors declare no conflicts of interest.

## Supporting information

Supporting Information
