# Supplementary material for: A peptide‐display protein scaffold to facilitate single molecule force studies of aggregation‐prone peptides
Source: Protein Sci. 2018 Mar 10;27(7):1205–17. doi: 10.1002/pro.3386 (PMC6032367; doi:10.1002/pro.3386)
Supplement: Supplementary file 1 — Supporting Information [file PRO-27-1205-s001.pdf]

Supplementary Material

Supplementary figures

```
         10         20         30
....|....| ....|....| ....|....|
MEEVTIKANL IFACGSTQTA EFKGTFEKAT

         40         50         60
....|....| ....|....| ....|....|
SEAYAYADTL KKDNGEWTVD VADKGGGARG

         70         80         90
....|....| ....|....| ....|....|
SVTGVTAVAQ KTVGSARGGG YTLNFKFAGS

....|..
GHHHHHH 97
```

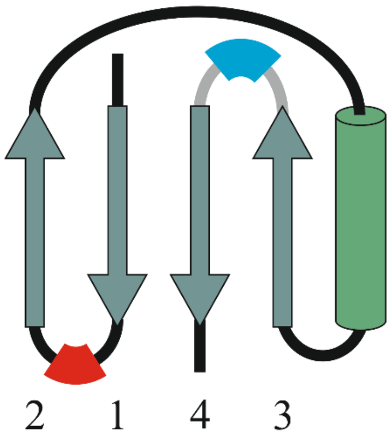

Figure S1. Sequence and topology of pL αSyn$_{71\text{-}82}$.

Left: primary sequence of pL αSyn$_{71\text{-}82}$. The sequences and position of the β3-β4 loop extension and the inserted αSyn$_{71\text{-}82}$ peptide are highlighted in grey and blue, respectively. Peptide sequences for γSyn$_{71\text{-}82}$ and the non-aggregation control $(GS)_6$ were inserted at the same position as αSyn$_{71\text{-}82}$. The engineered cysteine residue used for immobilisation is highlighted in red. Yellow highlighted text indicates the presence of a $(His)_6$ tag to enable Ni^{2+}-affinity purification. Right: structural schematic showing the position of the engineered cysteine residue used for immobilisation (red) relative to the position of the guest peptide (blue) with the extended β3-β4 loop (pale grey). Extension by the blue and red regions would shear the mechanically strong interface (β-strands 1 and 4).

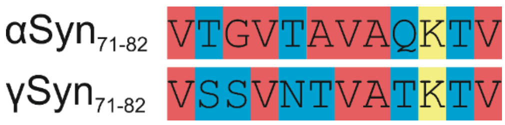

Figure S2. Sequence alignment of the central NAC regions of α- and γ-synuclein.

Residue properties are colour coded: red (hydrophobic), blue (polar) and yellow (charged). The sequences are highly homologous, 7/12 residues are identical and 3 of the 5 substitutions are conservative.

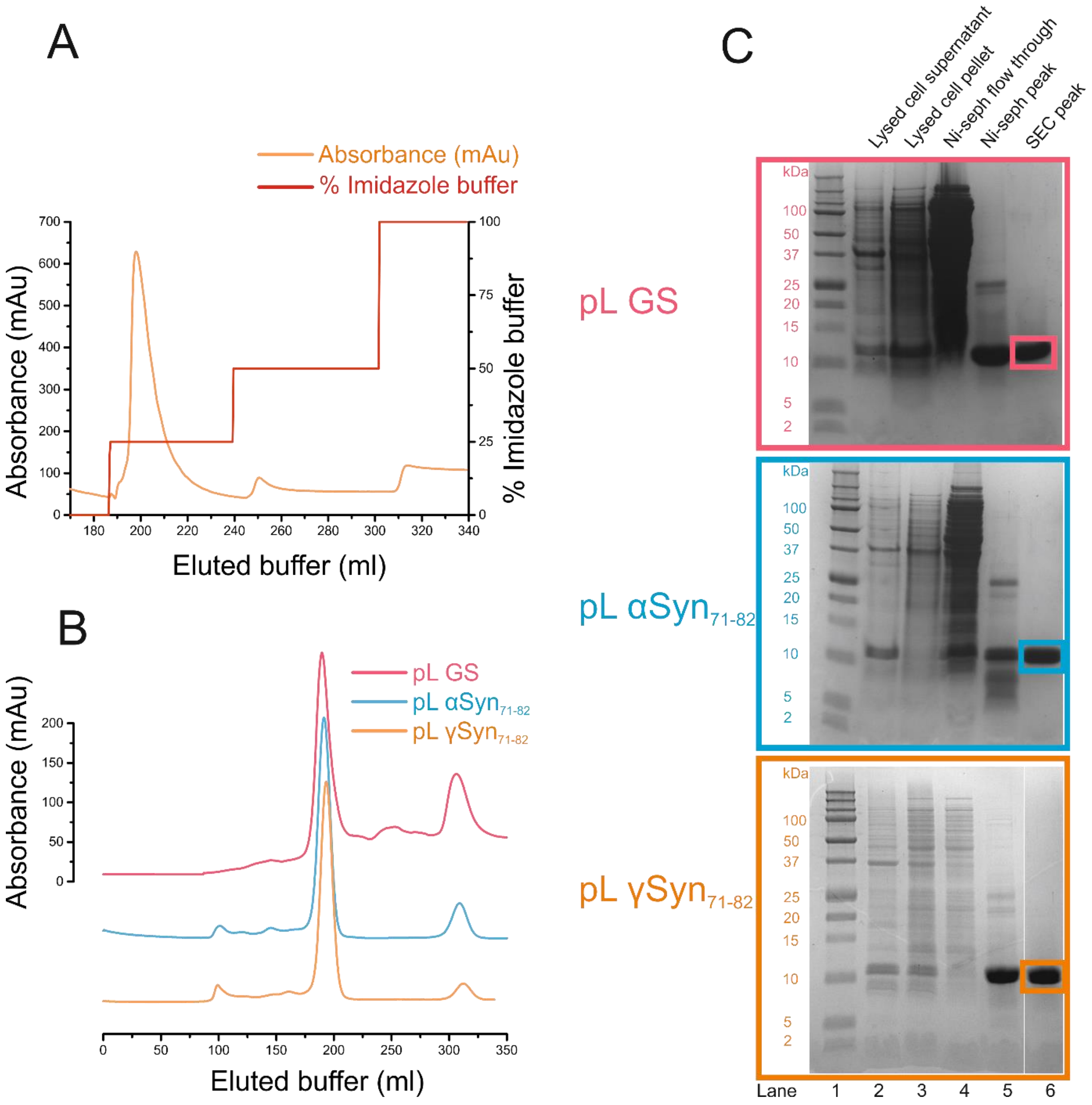

Figure S3. Two-step purification of pL variants.

(A) Typical elution profile for the Ni^{2+}-Sepharose affinity chromatography step (pL γSyn_{71-82}). The protein eluted in 25 % imidazole is visualised in the fifth lane in each gel in (C). (B) Elution profiles of the size exclusion chromatography step (SEC). The final purified protein after SEC is shown in lane 6 of the gels shown in (C), highlighted with a coloured box. (C) SDS-polyacylamide gels following the purification of pL GS (pink), pL αSyn_{71-82} (blue) and pL γSyn_{71-82} (orange).

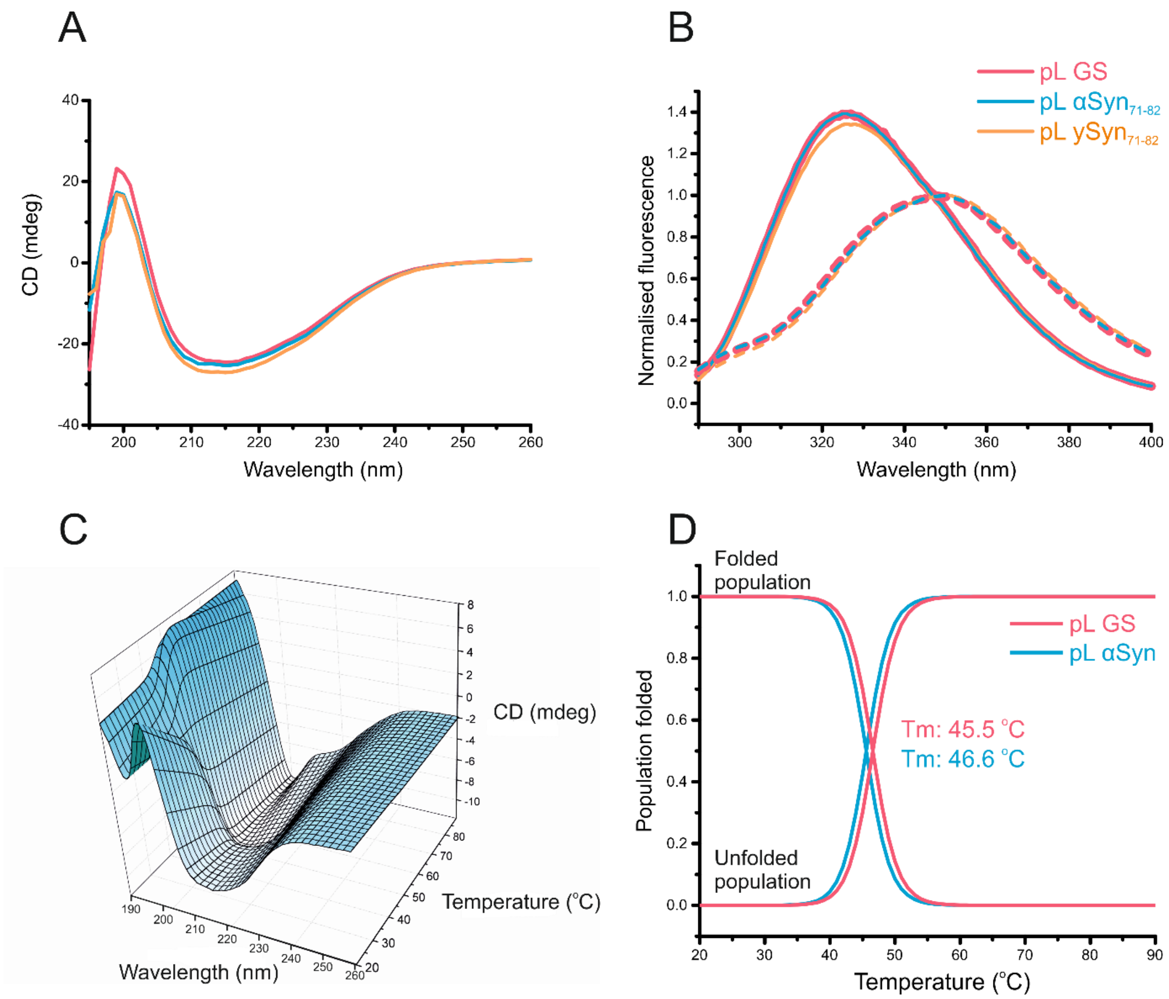

Figure S4. Spectroscopic analyses of pL variants.

(A) Far-UV CD spectra of 50 µM pL GS (pink); pL αSyn_{71-82} (blue) and pL γSyn_{71-82} (orange). The spectra are similar, exhibiting broad minima from ~210-220 nm, consistent with the mixed α/β topology of folded pL. (B) Intrinsic tryptophan fluorescence emission spectra of pL GS (pink), pL αSyn_{71-82} (blue) and pL γSyn_{71-82}, (orange) in the absence (solid lines) or presence of 8M urea (dashed lines). (C) Far UV-CD spectra as a function of temperature for pL αSyn_{71-82}. (D) The relative populations of folded and unfolded conformations of pL αSyn_{71-82} and pL GS (blue and pink respectively) calculated from the global analysis of CD thermal melt data. The T_m values are shown inset.

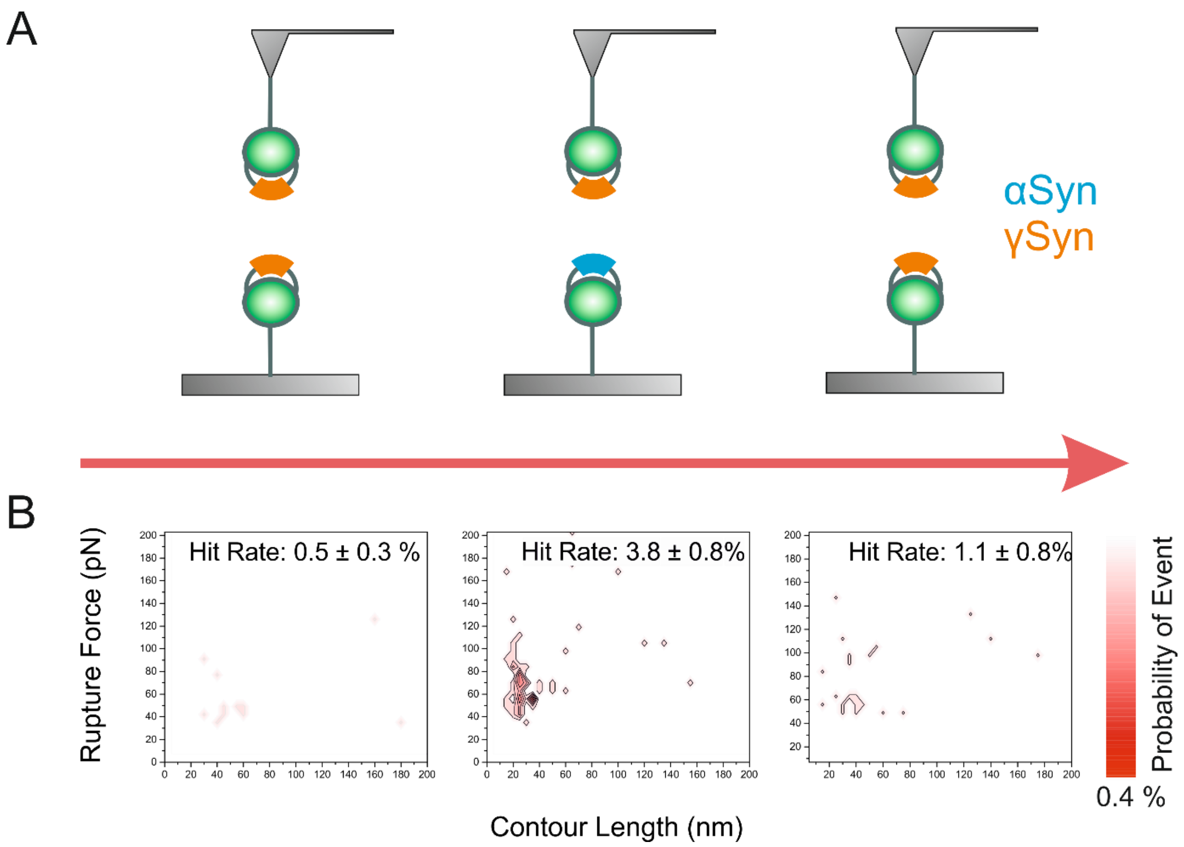

Figure S5. SMFS experiment showing presence of interaction-competent pL γSyn$_{71\text{-}82}$ on the AFM tip.

(A) Schematic of experimental setup. First the frequency of dissociation of pL γSyn$_{71\text{-}82}$ dimers (hit rate) was measured. The surface was then changed to one derivatised with pL αSyn$_{71\text{-}82}$ and the frequency of heterodimer dissociation measured. This sample was then replaced by the original pL γSyn$_{71\text{-}82}$ functionalised surface and the dissociation frequency measured once more. (B) Contour plots and calculated hit rates from the experiments described in (A). The intra-experimental errors between force maps are shown. The total number of approach retract cycles was 1500 for each different tip and surface pair.

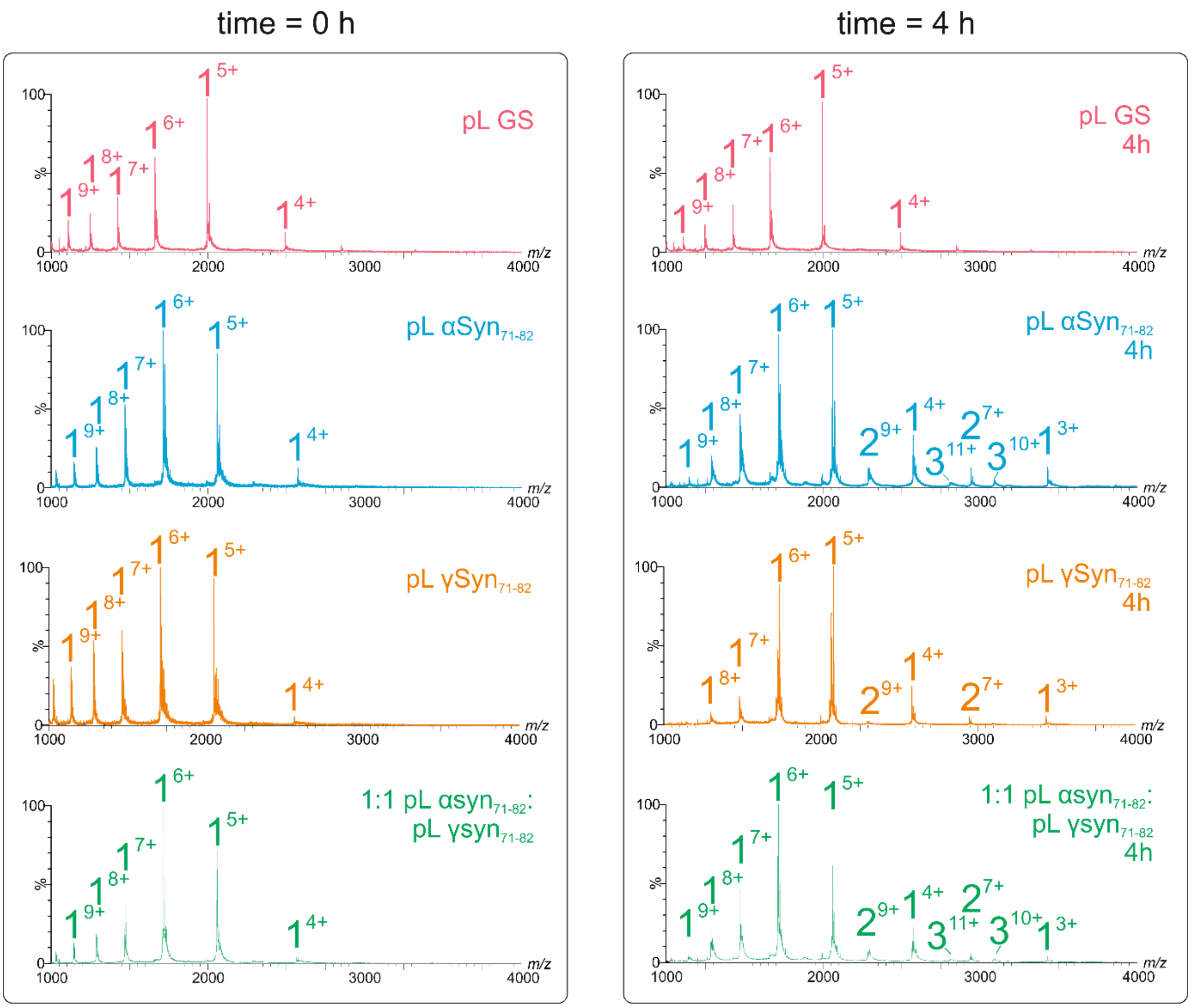

Figure S6. Native ESI-mass spectra of pL constructs immediately after dilution (t = 0) and after 4 h.

ESI-mass spectra of pL GS (pink), pL αSyn$_{71-82}$ (blue), pL γSyn$_{71-82}$ (orange) and a 1:1 mix of pL αSyn$_{71-82}$ and pL γSyn$_{71-82}$ (green) at t= 0 (left) and t = 4 h (right). The numbers above the peaks denote the oligomer order, with the positive-charge state of ions in superscript. All variants in isolation and a 1:1 mixture of pL αSyn$_{71-82}$ and pL γSyn$_{71-82}$ were present as monomer at t = 0 (left panel). After 4 h, all variants except the non-aggregating pL GS construct showed self-association (right panel). All samples were diluted to a final total protein concentration of 100 µM in 100 mM ammonium acetate buffer, pH 6.8.

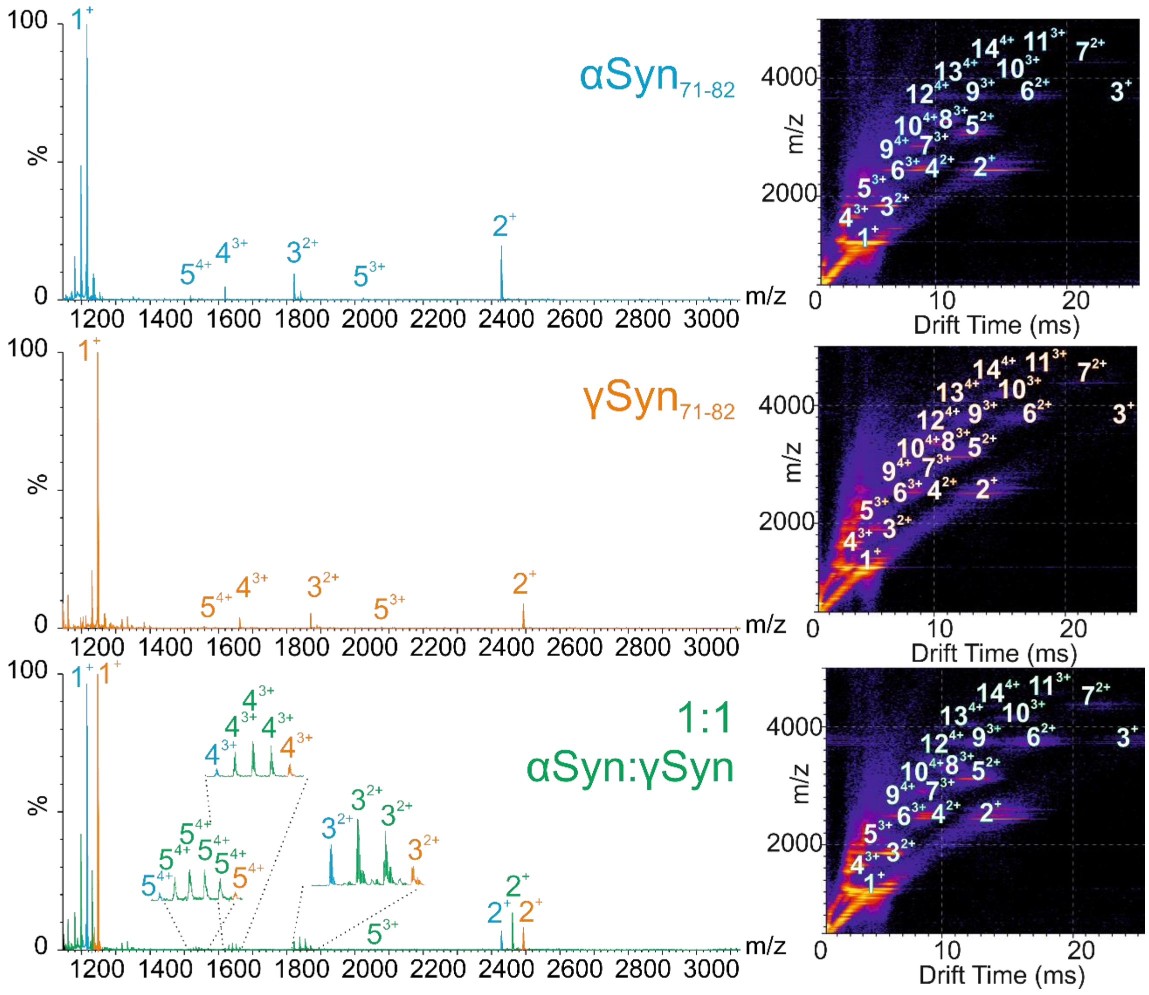

Figure S7. Mass spectrometric analyses of αSyn$_{71\text{-}82}$ and γSyn$_{71\text{-}82}$ peptides.

ESI mass spectra (left) and ESI IMS-MS driftscope plots (right) of αSyn$_{71\text{-}82}$ (top), γSyn$_{71\text{-}82}$ (middle) and a 1:1 mix of αSyn$_{71\text{-}82}$ and γSyn$_{71\text{-}82}$ (bottom). The numbers above the peaks denote the oligomer order, with the positive-charge state of ions in superscript. All mass spectra (left) confirm self-association of the peptides up to pentamer, with the 1:1 mix showing a random distribution between the number of αSyn$_{71\text{-}82}$ and γSyn$_{71\text{-}82}$ monomers in the oligomers. ESI–IMS–MS Driftscope plots (right) show monomeric to tetradecameric species present in all of the samples, two minutes after diluting the monomer to a final peptide concentration of 100 µM in 100 mM acetate buffer pH 6.8. ESI–IMS–MS Driftscope plots show the IMS drift time versus mass/charge (m/z) versus intensity (z, square-root scale).

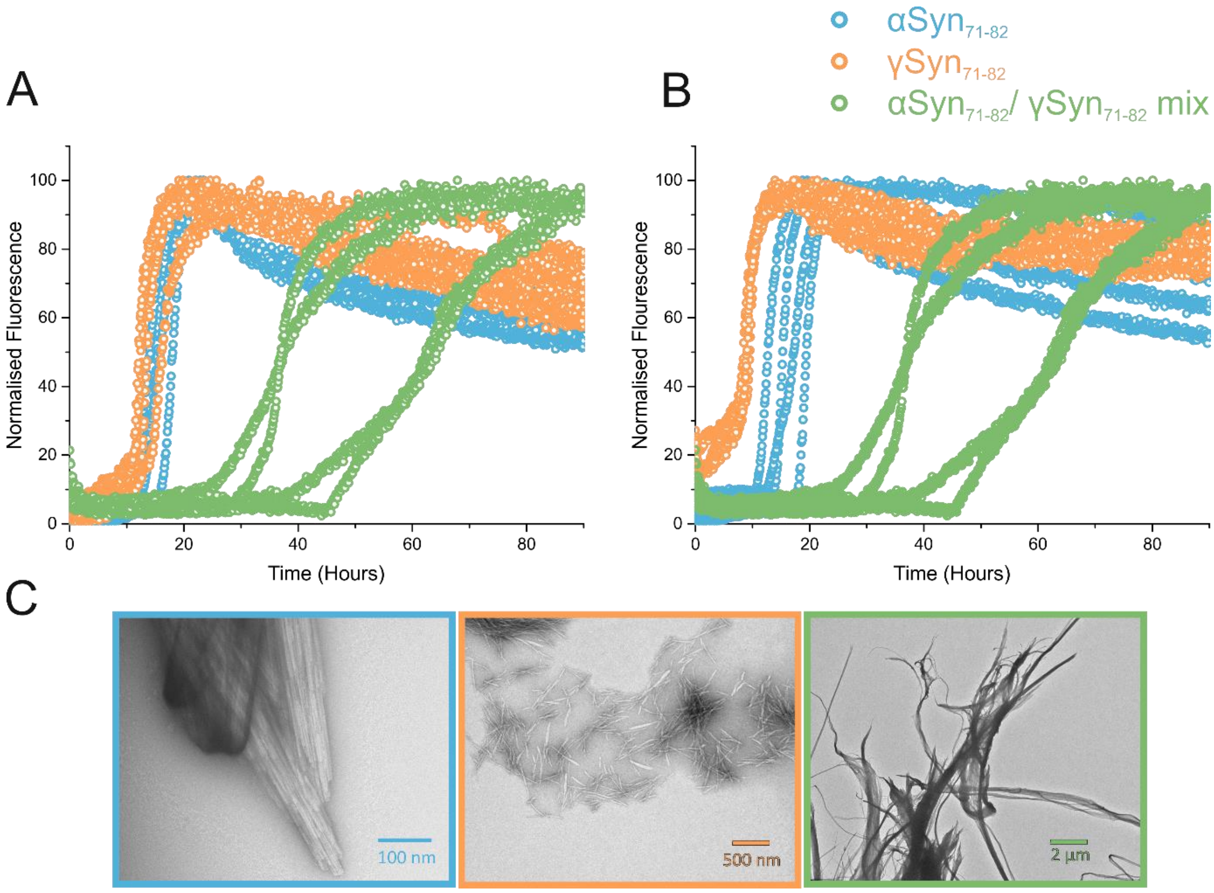

Figure S8. ThT fluorescence assay of αSyn$_{71\text{-}82}$ and γSyn$_{71\text{-}82}$ synthetic peptides. (A) Normalised fluorescence signal over time of 225 µM αSyn$_{71\text{-}82}$ (blue), 225 µM γSyn$_{71\text{-}82}$ (orange) and the 1:1 mix of αSyn$_{71\text{-}82}$ and γSyn$_{71\text{-}82}$ peptides (both at 225 µM, green). The lag times for αSyn$_{71\text{-}82}$, γSyn$_{71\text{-}82}$ and for the αSyn$_{71\text{-}82}$:γSyn$_{71\text{-}82}$ mixed incubation are 13.2 ± 2.1, 11.8 ± 1.2 and 34.6 ± 8.5 h, respectively. (B) Normalised fluorescence signal over time of 450 µM αSyn$_{71\text{-}82}$, 450 µM γSyn$_{71\text{-}82}$ and the 1:1 mix of αSyn$_{71\text{-}82}$ and γSyn$_{71\text{-}82}$ peptides (both at 225 µM giving a final peptide concentration of 450 µM). Colours denote the same peptides as described in (A). Note: the αSyn$_{71\text{-}82}$ + γSyn$_{71\text{-}82}$ mix is the same data as presented in (A). The lag times for αSyn$_{71\text{-}82}$ and γSyn$_{71\text{-}82}$ are 13.3 ± 3.2 and 6.1 ± 1.0 h, respectively. (C) TEM images taken at the end points (100 h) of the incubations (colour coded as above). All three incubations form fibrillary structures, but with notably different morphology.

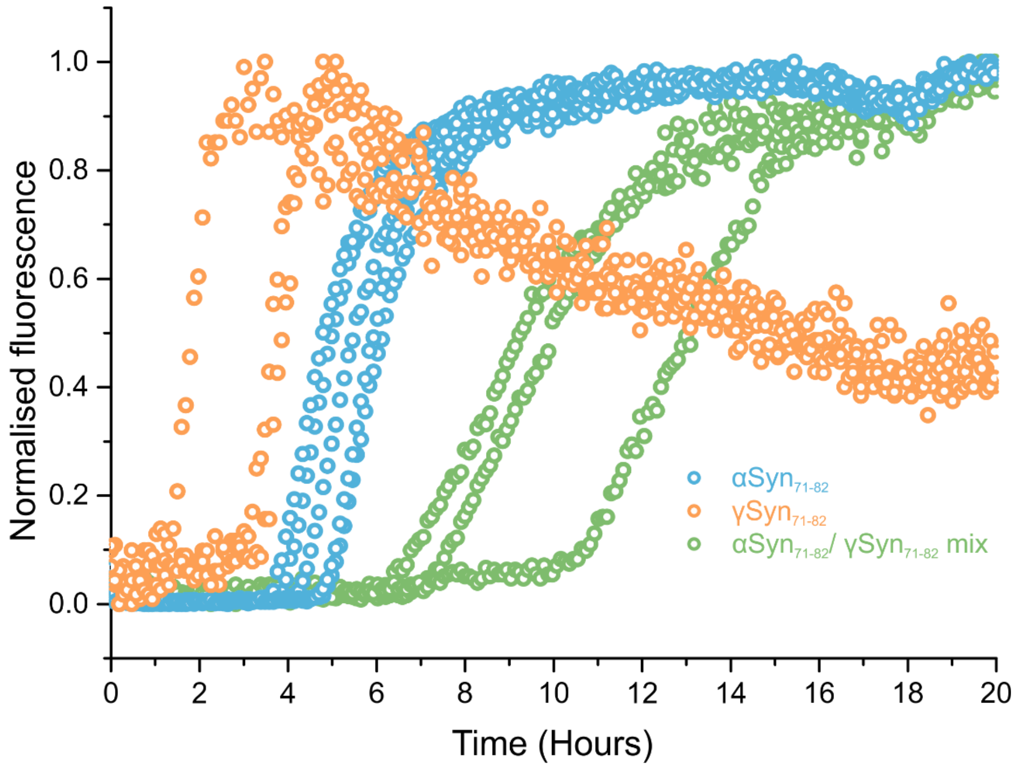

Figure S9. ThT fluorescence assay of $\alpha Syn_{71\text{-}82}$ and $\gamma Syn_{71\text{-}82}$ synthetic peptides in ESI-MS buffer conditions. Normalised fluorescence signal over time of 225 µM $\alpha Syn_{71\text{-}82}$ (blue), 225 µM $\gamma Syn_{71\text{-}82}$ (orange) and the 1:1 mix of $\alpha Syn_{71\text{-}82}$ and $\gamma Syn_{71\text{-}82}$ peptides with a total peptide concentration of 225 µM (both at 112 µM, green). The lag times for $\alpha Syn_{71\text{-}82}$, $\gamma Syn_{71\text{-}82}$ and for the $\alpha Syn_{71\text{-}82}$:$\gamma Syn_{71\text{-}82}$ mixed incubation are 4.2 ± 0.6, 2.5 ± 1.1 and 7.8 ± 2.0 h, respectively. ThT experiments presented here were carried out in 100 mM ammonium acetate, pH 6.8 (in line with the ESI-MS experimental conditions).

Supplementary Tables

Protein	Expected mass Da	Observed mass Da	Mass difference (Da)
pL GS	10 100	9 967 ± 3	-133
pL αSyn$_{71-82}$	10 390	10 257 ± 4	-132
pL γSyn$_{71-82}$	10 423	10 290 ± 4	-133

Supplementary Table 1. Observed and expected masses from ESI-MS data. The observed mass is 133 Da below the expected mass as the N-terminal Met residue is excised, due to the activity of the *E. coli* enzyme methionyl-aminopeptidase which has increased activity with decreasing residue size in the penultimate N-terminal position (alanine in this case)(1). Errors are quoted to the nearest Dalton.

References

1. Hirel PH, Schmitter JM, Dessen P, Fayat G, Blanquet S (1989) Extent of N-terminal methionine excision from *Escherichia coli* proteins is governed by the side-chain length of the penultimate amino-acid. *Proc Natl Acad Sci USA* **86**:8247-8251.
